# Hypertriglyceridemia-Induced Pancreatitis Complicated by Diabetic Ketoacidosis

**DOI:** 10.7759/cureus.19985

**Published:** 2021-11-29

**Authors:** Syed Mujtaba Ali Naqvi, Syed Haider, Anup Patel, Shoaib Muhammad, Amman Yousaf

**Affiliations:** 1 Internal Medicine, University of Kentucky, Bowling Green, USA; 2 Urology, Al-Aleem Medical College, Gulab Devi Hospital, Lahore, PAK; 3 Radiology, Salam Medical Complex, Lahore, PAK; 4 Internal Medicine, McLaren Flint, Michigan State University, Flint, USA

**Keywords:** diabetic ketoacidosis, insulin pump, hypertriglyceridemia-induced acute pancreatitis, pancreatitis causes, severe hypertriglyceridemia

## Abstract

Acute pancreatitis is a potentially fatal clinical entity having multiple underlying triggers. Though the incidence of hypertriglyceridemia-induced pancreatitis is low; however, patients with such risk factors develop severe disease. We present a case of a 47-year-old male who came to our facility with complaints of epigastric pain. Physical examination and laboratory workup unmasked the presence of pancreatitis alongside concurrent diabetic ketoacidosis (DKA). This presentation is unique, and to our knowledge, only a few cases have been reported in the literature. Furthermore, the co-existence of pancreatitis and DKA can overlap the clinical picture of each other, which might lead to unwanted complications if not diagnosed timely.

## Introduction

Acute pancreatitis (AP) is a life-threatening condition and a significant cause of acute abdominal pain. The incidence approaches up to 73 cases per 100,000; however, in the United Kingdom, the incidence range is 15-42 patients per 100,000 [1]. Out of a few crucial underlying etiologies, hypertriglyceridemia (HTG) poses a significant risk of developing severe disease. AP related to this pathology usually presents in predisposed patients having genetic lipoprotein abnormality. Such cases count around 2%-4% of all AP diagnoses. But HTG-induced pancreatitis is particularly important as it is associated with far more complications due to the severity [2]. We report a case of a 47-year-old male patient who presented with epigastric pain. The workup unmasked the presence of HTG-induced pancreatitis on the background of diabetic ketoacidosis (DKA). It is quite a rare presentation, and to our knowledge, only a few cases have been reported in the literature. Our article also emphasizes the importance of early diagnosis and management, as both AP and DKA can worsen each other.

## Case presentation

A 47-year-old male was transferred to the emergency department from a primary care facility with complaints of epigastric abdominal pain. In addition, his pain was associated with nausea and vomiting. On examination, the patient was in mild distress, and he was irritable. Palpation appreciated mild tenderness in the epigastric region; however, there was no guarding or rebound tenderness. His family history was insignificant for any lipid abnormalities, diabetes, or cardiac disease. His laboratory investigations revealed changes in blood glucose and bicarbonate, and urinalysis showed urinary ketones consistent with diabetic ketoacidosis. Significant laboratory value are summarised in Table 1.

**Table 1:** Laboratory investigations at the time of presentation

Parameters	Specimen	Value	Reference values
Triglycerides	Serum	8075	<150 mg/dL
Blood glucose random	Serum	278	72-99 mg/dL
Serum lipase	Serum	5205	0-50 U/L
Serum bicarbonate	Serum	9	23-30 mEq/L
Urinary ketones	Urine	4+	Nil

In line with increased triglycerides and lipase, a provisional diagnosis of hypertriglyceridemia-induced acute pancreatitis was made. To further evaluate, an urgent abdominal ultrasound was requested on which the pancreas couldn't be visualized due to overlying gassy bowel loops.

**Figure 1:**
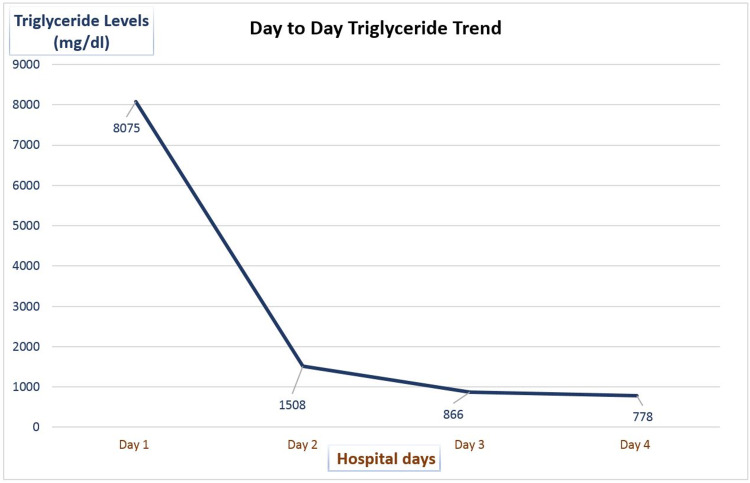
Levels of triglycerides against days of management

The patient was immediately admitted to the intensive care unit (ICU) and aggressive fluid resuscitation was done along with adequate analgesia and nutritional support. Parallel running DKA was managed with intravenous insulin with fluids and electrolytes replacement. He was also prescribed tablet fenofibrate 160 mg daily and tablet icosapent ethyl 2 g twice a day for overtly increased triglycerides. On the second day, the patient reported significant relief from abdominal pain, and his nausea and vomiting also settled. Laboratory investigations revealed a remarkable decrease in triglycerides to be 1508 mg/dL (Figure 1).

In addition, the anion gap closed back to normal, and metabolic acidosis resolved. Consequently, his insulin drip was stopped, and he was started on a combination of insulin glargine and lispro. He showed further improvements clinically, and his triglyceride level plummeted to 866 mg/dL on the next day. After the 36-hour observance in the ICU, the patient was safely discharged on Tricor (fenofibrate), Vascepa (icosapent ethyl), atorvastatin alongside insulin Lantus (glargine) and Humalog (lispro). On one- and two-week follow-up visits, he showed no signs of relapse and is healthy to date.

## Discussion

AP is a life-threatening inflammatory condition. After cholelithiasis and alcohol, HTG is the third most common trigger. Other noteworthy causes include direct pancreatic injuries, impaired secretion, and abnormally activated enzymes causing autodigestion of the pancreas. AP presents as severe abdominal pain, and laboratory investigations show elevated pancreatic enzymes in the blood.

HTG becomes a significant risk factor when the triglyceride level goes over 1000 mg/dL. Various pathways are thought to play a part in the development of pancreatitis. Triglycerides are broken down into free fatty acids (FFAs) with the action of pancreatic lipases. Within the normal limits, these FFAs are bound by plasma proteins. In HTG, a significantly larger number of FFAs are released into the blood, outweighing plasma proteins' binding capacity. These unbound FFAs tend to bind together, forming smaller structures known as micelles. Due to their detergent properties, these micelles can damage the blood products and vessels, causing ischemia of the pancreas. Overtly increased FFAs also alter the blood properties like viscosity that ultimately leads to capillary blockage [3]. Consequently, ischemia of the pancreas results due to the loss of blood flow. Ischemia can eventually cause the activation of pancreatic enzymes by creating an acidic environment leading to autodigestion of the pancreas.

DKA is another potentially fatal state that results from a deficiency of insulin [4]. This causes decreased lipoprotein lipase activity, thereby worsening HTG. In type 2 diabetes, insulin resistance causes upregulation of triglyceride production. Concurrently, HTG-induced pancreatitis results in the reduced production of insulin in pancreatic beta cells [5]. Therefore, both pancreatitis and DKA overwhelm each other and worsen the clinical picture.

Laboratory investigations play a vital role in the diagnosis and management of pancreatitis and DKA. The diagnosis of AP is based on the revised Atlanta classification criteria, which requires two out of the three conditions to be met: (i) acute onset of severe epigastric pain usually radiating to back, (ii) increased serum lipase and amylase to three times or more, (iii) findings of AP on imaging studies [6]. For HTG to be considered a culprit, a level of around 1000 mg/dL is needed. Our patient had two out of the three criteria for a diagnosis of AP. Furthermore, he had severely raised triglycerides to be considered the culprit. Similarly, DKA was diagnosed based on the laboratory findings of hyperglycemia and decreased bicarbonate levels.

Fluid resuscitation plays a pivotal role in managing both conditions, similar to the treatment of our patient. DKA was treated with intravenous insulin, and his electrolytes were normalized. Although no set guidelines on managing HTG-induced pancreatitis are developed, it is prudent to lower triglyceride levels. For this purpose, various strategies and medications are being used. We managed our patient with tablet Tricor (fenofibrate) and Vascepa (icosapent ethyl), and he showed excellent improvement in three days. Treating the concurrent DKA is also crucial, as decreased insulin causes a rise in the triglyceride level in the blood, creating a hindrance in the management of HTG. The use of insulin in such cases is bifold, as it manages DKA while also improving HTG by activating lipoprotein lipase [7].

## Conclusions

Patients presenting with a suspected diagnosis of AP should also be evaluated for possible DKA and vice versa. As both the conditions worsen each other, it is crucial to manage them side by side. Laboratory investigations are vital in diagnosing, and imaging studies are critical in visualizing AP. Fluids play a pivotal role in managing HTG-induced AP as well as DKA. Triglyceride-lowering agents like fenofibrate are needed for HTG, while DKA should be treated with intravenous insulin.
